# Global burden of diabetes mellitus 1990–2021: epidemiological trends, geospatial disparities, and risk factor dynamics

**DOI:** 10.3389/fendo.2025.1596127

**Published:** 2025-07-01

**Authors:** Ciming Pan, Beiling Cao, Hui Fang, Yelu Liu, Shuhan Zhang, Wei Luo, Yuanjie Wu

**Affiliations:** ^1^ Hefei Xinzhuan High-tech Zone Huoshuiyuan Traditional Chinese Medicine Clinic, Outpatient Department, Hefei, Anhui, China; ^2^ Nanjing University of Chinese Medicine, First Clinical Medical College, Nanjing, Jiangsu, China; ^3^ College of Traditional Chinese Medicine, Anhui University of Chinese Medicine, Hefei, Anhui, China; ^4^ Department of Traditional Chinese Acupuncture and Tuina, the Second People's Hospital of Anhui Province, Hefei, Anhui, China; ^5^ Pediatric Neurorehabilitation Center, the First Affiliated Hospital of Anhui Medical University, Hefei, Anhui, China; ^6^ School of Basic Medical Sciences, Yunnan University of Traditional Chinese Medicine, Kunming, Yunnan, China

**Keywords:** diabetes mellitus, global burden of disease, disability-adjusted life years, geospatial disparities, risk factors

## Abstract

Diabetes mellitus(DM) remains a significant global health challenge, characterized by high incidence and Disability-adjusted life years (DALYs) rates. A comprehensive understanding of the disease burden of DM is crucial for developing effective prevention and treatment strategies. This study analyzes the global burden of diabetes mellitus (DM) from 1990 to 2021 using data from the Global Burden of Disease Study. In 2021, DM caused 1.66 million deaths, with age-standardized mortality rising by 7.95% since 1990. While Type 1 diabetes (T1DM) mortality declined by 29.24%, Type 2 diabetes (T2DM) accounted for 97.1% of deaths, with a 9.75% mortality increase. Geospatial analysis revealed extreme disparities: Pacific Island nations exhibited incidence rates >800/100,000, contrasting with <3/100,000 in Costa Rica and China. T1DM burden predominated in high-latitude regions (e.g., Finland), whereas T2DM mirrored overall DM distribution. DALYs for T2DM surged by 42.32%, disproportionately affecting Low-SDI regions. Age-stratified analyses identified distinct patterns: T2DM incidence peaked at 59–69 years, while T1DM DALYs peaked in early-middle adulthood (40–44 years). Risk factor hierarchies highlighted metabolic risks (e.g., high BMI) for T2DM and ambient temperature effects for T1DM. Despite progress in T1DM management, rising T2DM burden underscores urgent need for targeted prevention strategies addressing obesity, dietary risks, and environmental determinants. Study limitations include potential GBD data inaccuracies and ecological design constraints. These findings emphasize the imperative for region-specific interventions to mitigate the evolving diabetes pandemic.

## 1 Introduction

Diabetes mellitus has emerged as one of the most pressing global public health challenges of the 21st century (1). This metabolic disorder, characterized by chronic hyperglycemia, not only directly damages multiple organ systems but also exacerbates the burden of cardiovascular diseases, renal failure, and neuropathies (2). Despite significant advances in clinical management, the International Diabetes Federation projects that 783 million people will be living with diabetes by 2045—a trajectory that threatens to overwhelm healthcare systems and impede progress toward Sustainable Development Goals related to non-communicable diseases.

The urgency to address this epidemic is underscored by its disproportionate socioeconomic impact. According to GBD data, stark disparities are evident: low- and middle-income countries now bear 80% of the diabetes burden, yet lack the infrastructure for early diagnosis and affordable insulin (3, 4). Meanwhile, rising obesity rates and sedentary lifestyles are driving an increase in type 2 diabetes in high-income nations, costing economies approximately $1.3 trillion annually in direct medical expenditures and lost productivity (5). Current interventions, however, remain fragmented. Most epidemiological studies focus on national-level trends, neglecting subregional variations in risk factors such as air pollution, dietary shifts, and genetic predispositions—variations that GBD’s granular data could elucidate (6).

This study leverages the GBD database to address critical gaps. First, we analyze three-decade trends in diabetes incidence, mortality, and disability-adjusted life years (DALYs) across 204 countries, identifying under-researched hotspots where prevalence defies global patterns. Second, we quantify the attribution of modifiable risks (e.g., high body mass index [BMI], poor diet) using GBD’s comparative risk assessment framework, enabling targeted prevention strategies. Finally, we model the long-term cost of inaction, emphasizing how demographic aging and climate change-related food insecurity may accelerate the crisis. By synthesizing GBD’s multidimensional data, this paper aims to equip policymakers with evidence to prioritize context-specific interventions, thereby mitigating a preventable catastrophe.

## 2 Methodologies and materials

### 2.1 Data extract

Utilized the latest publicly available GBD 2021 dataset, covering diabetes-related metrics (incidence, prevalence, mortality, disability-adjusted life years across 204 countries and territories from 1990 to 2021. Included data on diabetes subtypes: T1DM and T2DM.

### 2.2 Disease burden metrics

Calculated age-standardized rates (per 100,000 population) for incidence, prevalence, mortality, and DALYs using the GBD reference population.

Estimated DALYs as the sum of years of life lost (YLLs) and years lived with disability (YLDs), weighted by disability weights specific to diabetes complications (e.g., neuropathy, retinopathy).

Used Spatio-Temporal Gaussian Process Regression (ST-GPR) to smooth trends and predict values for regions with sparse data.

### 2.3 Risk factor attribution

Quantified population-attributable fraction for modifiable risk factors (e.g., high BMI) using comparative risk assessment frameworks.

Uncertainty Analysis: Reported 95% uncertainty intervals (UIs) generated through 1,000 Monte Carlo draws at each computational step.

### 2.4 Stratified analyses

Disaggregated results by age (10-year age groups), sex, Socio-Demographic Index (SDI) quintiles, and geographic regions (GBD super-regions). Compared trends between high-income countries and low-middle-income countries.

### 2.5 Statistical analysis methods

Analyses were performed using the GBDR_V2.36 software (https://medhub.shinyapps.io/ShinyGBD/) to: Quantify the contributions of age, period, and cohort effects to changes in anxiety disorder burden. Integrate historical trends and demographic shifts to project anxiety disorder burden from 2022 to 2035. Conduct visualization analysis. All statistical analyses and data visualizations were performed using R (version 4.3.3) and JD_GBDR (V2.24, Jingding Medical Technology Co., Ltd.).

BAPC Models for Projections: (Bayesian Age-Period-Cohort) models provide a comprehensive framework for making projections using integrated nested Laplace approximations (INLA) for full Bayesian inference. Key features of BAPC models include: 1.Generation of age-specific and age-standardized projected rates. 2.Automatic addition of Poisson noise when interest lies in the predictive distribution. BAPC is particularly useful for projecting future rates based on historical data, making it an invaluable tool for public health planning and analysis(Analysis Tools Help Page (https://folk.ntnu.no/andrerie/software.html). For detailed parameters and explanations, refer to the article (PMID: 28139001).

## 3 Results

### 3.1 DM burden in 2021

**Figure 1:**
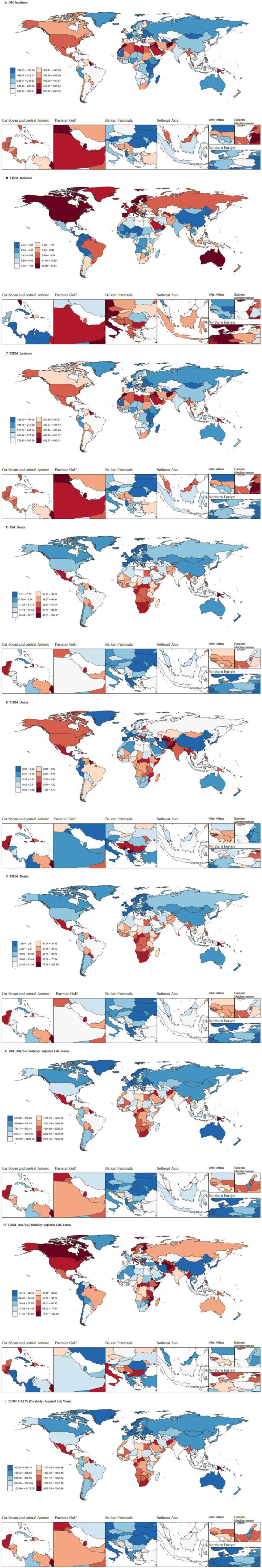
Age-standardized incidence rate DALYs rate and death rate in 204 countries and territories in 2021.

As shown in **Figure 1** and **Supplementary Table 1**, The global burden of diabetes mellitus demonstrated significant epidemiological shifts between 1990 and 2021. In 2021, diabetes accounted for 1,656,634 deaths worldwide, with an age-standardized death rate (ASDR) of 19.61 per 100,000 population (95% UI: 18.12-20.83). Compared to 1990 baseline data, this represents a 7.95% increase in mortality rate, accompanied by a 71.37% surge in incidence and 38.18% rise in DALY rate. Subtype analysis revealed divergent tren ds: T1DM showed encouraging progress with 29.24% mortality reduction and 12.00% DALY decrease since 1990, despite a 22.80% incidence increase (48,511 deaths; ASDR 0.59, 95% UI: 0.53-0.66). Conversely, T2DM accounted for 97.1% of total diabetes mortality (1,608,123 deaths; ASDR 19.02, 95% UI: 17.57-20.20), demonstrating 9.75% mortality increase and 42.32% DALY growth alongside 73.08% incidence escalation.

Geospatial analysis uncovered striking disparities in age-standardized incidence rates (ASIR). Pacific Island nations dominated global rankings, with Marshall Islands (884.21; 953.65-825.84), American Samoa (863.36; 926.74-803.32), and Fiji (838.06; 872.60-803.64) exhibiting the highest DM incidence - rates exceeding 800/100,000 compared to <3/100,000 in lowest-ranking Costa Rica (2.37; 2.86-1.97), China (2.67; 3.26-2.21), and Colombia (2.68; 3.18-2.26). T1DM distribution showed distinct northern latitude predominance, with Finland (44.40; 46.81-42.24), Canada (35.24; 37.90-32.85), and Italy (26.70; 33.34-21.92) leading incidence rankings, while T2DM spatial patterns mirrored overall DM distribution.

Mortality burden analysis revealed Fiji as the epicenter of diabetes-related deaths across all categories, recording extreme ASDR values of 266.11 (329.40-213.67) for general DM, 265.22 (328.29-213.01) for T1DM, and 265.22 (328.29-213.01) for T2DM - rates over 5,000% higher than Singapore’s record-low 0.047 (0.05-0.04) for general DM. DALY burden analysis further emphasized the Pacific Islands’ disproportionate impact, with Fiji demonstrating 7,387.94 (9,157.60-5,995.40) DALYs for general DM and 7,322.65 (9,075.98-5,942.63) for T2DM, contrasting sharply with European nations like France (354.54; 450.10-281.91) and Belarus (307.04; 397.01-237.08) showing minimal burden. Notably, T1DM-specific patterns revealed unexpected extremes, with Haiti (180.67; 262.98-115.85 DALYs) and Finland (120.43; 159.68-86.84) representing burden polarities despite geographical and developmental disparities.

### 3.2 Time trends of DM disease burden in regions with diverse SDI levels

**Figure 2:**
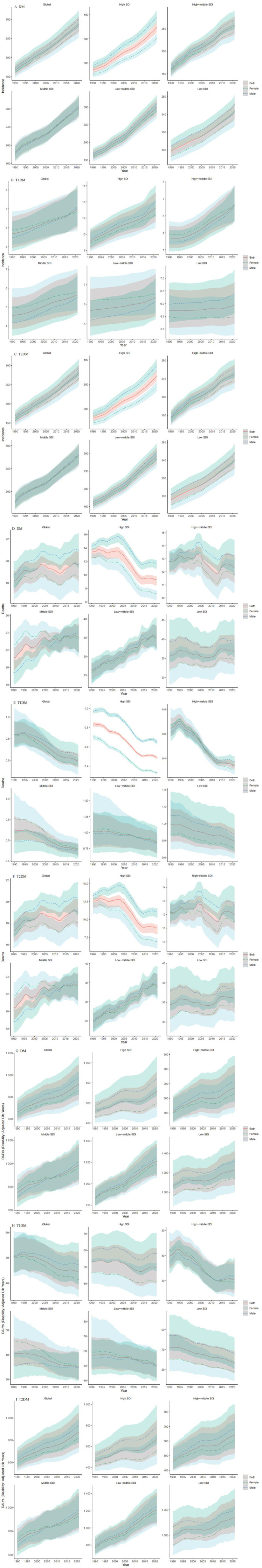
The incidence rate DALYs rate and death rate trend of different genders globally and in different SDI regions from 1990 to 2021.

From 1990 to 2021, the global incidence rates of diabetes mellitus (DM), type 1 diabetes (T1DM), and type 2 diabetes (T2DM) demonstrated sustained upward trajectories, with females exhibiting a more pronounced increase across all diabetes categories (**Figures 2A–C**). Demographic analysis revealed significant growth patterns in High-SDI regions, particularly for male DM cases, both genders with T1DM, and male T2DM patients. Middle-SDI regions showed marked increases in male DM and T2DM prevalence.

Disability-Adjusted Life Years (DALYs) analysis presented divergent patterns: T1DM-associated DALYs showed a global decline, with female patients demonstrating a steeper reduction trend (**Figures 2D–F**). Conversely, T2DM-related DALYs displayed an upward trajectory, particularly pronounced in male populations. Regionally, Low-SDI areas consistently demonstrated the highest DALY burdens across all diabetes types.

Geographic disparities were particularly evident in mortality patterns, with Global and Low-SDI regions sustaining elevated mortality rates compared to High-SDI and High-Middle SDI areas (**Figures 2G–I**). Longitudinal analysis revealed a significant 31-year decline in T1DM mortality rates (1990-2021), while T2DM mortality maintained a relatively stable trajectory with minimal fluctuations.

### 3.3 Age trends of DM disease burden by 2021

**Figure 3:**
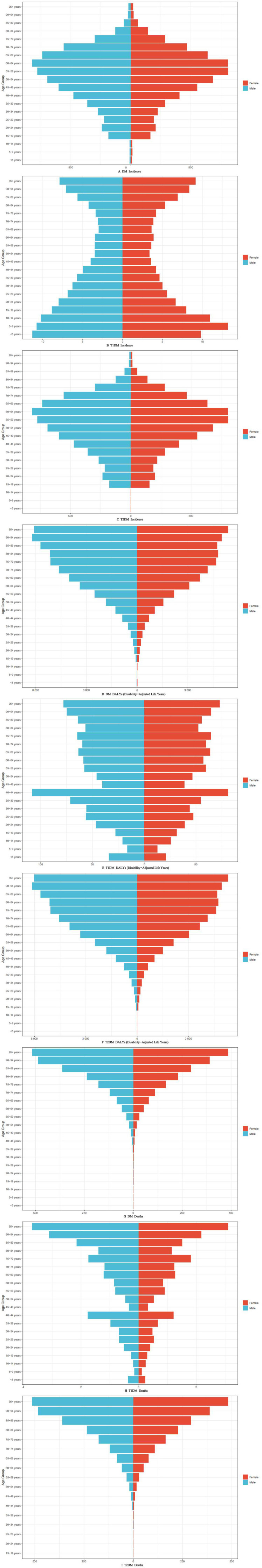
Age-standardized incidence rate DALYs rate and death rate worldwide in 2021.

**Figure 3** illustrates the 2021 age-stratified epidemiological patterns of diabetes mellitus (DM), type 1 diabetes (T1DM), and type 2 diabetes (T2DM) across incidence, Disability-Adjusted Life Years (DALYs), and mortality.

Incidence Distribution (**Figures 3A–C**): DM and T2DM exhibited spindle-shaped age-specific incidence profiles, peaking notably in the 59–69-year cohort. In contrast, T1DM demonstrated a bimodal distribution, characterized by disproportionately lower incidence rates among individuals aged 50–79 years compared to younger and older populations.

DALYs Burden (**Figures 3D–F**): For DM and T2DM, DALYs displayed a progressive escalation with advancing age, reflecting cumulative disease impacts. T1DM-associated DALYs, however, peaked sharply in the 40–44-year age group, suggesting heightened disability burdens during early-middle adulthood.

Mortality Trends (**Figures 3G–I**): Age-dependent mortality for DM and T2DM followed a monotonic increase aligned with aging. T1DM mortality exhibited a distinct biphasic pattern: a steady rise from ages 5–44 years, followed by a secondary acceleration phase between ages 45–95 years, highlighting differential risk dynamics across the lifespan.

### 3.4 DALYs risk analysis for diabetes mellitus

**Figure 4:**
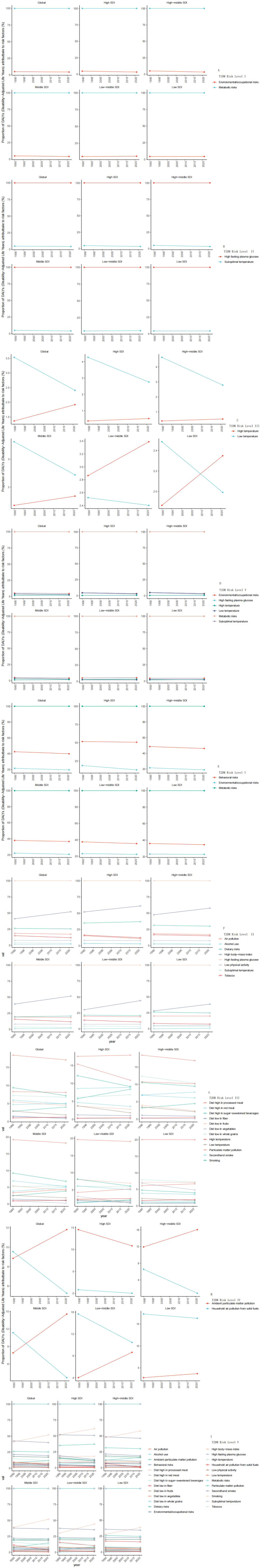
Risk factors for T1DM and T2DM DALYs worldwide in 2021.

The risk levels of T1DM were shown in **Figure 4A** (Risk factors of all levels). The hierarchical risk analysis revealed metabolic risks and environmental/occupational risks as primary (Level 1) contributors to T1DM-associated DALYs, with temporal stability observed from 1990 to 2021. At Level 2, suboptimal temperature and high fasting plasma glucose demonstrated minimal temporal variations. Notably, Level 3 analysis identified distinct thermal influences: low temperature constituted a predominant risk factor across Global, High SDI, High-middle SDI, Middle SDI, and Low-middle SDI regions in 1990, showing significant reductions (1990-2021) in all aforementioned geographical categories. Conversely, high temperature exhibited a global upward trajectory, particularly marked in Middle SDI and Low-middle SDI regions.

The risk levels of T2DM were shown in **Figure 4B** (Risk factors of all levels). The risk hierarchy identified metabolic risks, behavioral risks, and environmental/occupational risks as Level 1 determinants, maintaining stable temporal patterns throughout the study period. Level 2 analysis highlighted three modifiable factors: high fasting plasma glucose, elevated body mass index (BMI), and dietary risks, with BMI demonstrating a concerning upward trend. Level 3 examination revealed moderate declines in three specific risks: processed meat consumption, particulate matter pollution, and smoking. At Level 4, ambient particulate matter pollution showed divergent patterns - substantial increases in Global, High-middle SDI, Middle SDI, and Low-middle SDI regions contrasted with decreases in High SDI areas. Household air pollution from solid fuels displayed consistent global reductions.

## 4 Discussion

DM remains a cornerstone of global public health challenges, with its burden escalating significantly between 1990 and 2021. The data reveal a complex interplay of rising incidence, shifting mortality patterns, and growing DALYs, underpinned by subtype-specific trends (T1DM, T2DM), geospatial disparities, and socio-demographic influences. This discussion dissects these findings, interprets the data’s implications, and explores the scientific and societal factors driving these epidemiological shifts.

### 4.1 Global epidemiological shifts in diabetes burden (1990–2021)

#### 4.1.1 Mortality trends and subtype divergence

In 2021, DM accounted for 1,656,634 deaths globally, with an ASDR of 19.61 per 100,000 (95% UI: 18.12–20.83), marking a 7.95% increase from 1990. This rise contrasts sharply with subtype-specific trajectories: T1DM mortality declined by 29.24% (48,511 deaths; ASDR 0.59, 95% UI: 0.53–0.66), while T2DM, comprising 97.1% of DM deaths (1,608,123 deaths; ASDR 19.02, 95% UI: 17.57–20.20), increased by 9.75%. These divergent trends reflect distinct pathophysiological and management profiles.

The reduction in T1DM mortality likely stems from advancements in insulin therapy, continuous glucose monitoring, and structured patient education, which have improved glycemic control and reduced acute complications like diabetic ketoacidosis (7). Conversely, T2DM’s mortality surge aligns with its 73.08% incidence increase and 42.32% DALY growth, driven by aging populations, urbanization, and lifestyle factors such as sedentary behavior and obesogenic diets (8). The overwhelming dominance of T2DM in mortality underscores its status as a non-communicable disease (NCD) epidemic, necessitating targeted interventions.

#### 4.1.2 Incidence and DALY escalation

Global DM incidence rose by 71.37% from 1990 to 2021, with T2DM leading at 73.08% compared to T1DM’s 22.80%. The DALY rate increased by 38.18% overall, with T2DM’s 42.32% rise contrasting T1DM’s 12.00% decline. These metrics highlight a growing disease footprint, particularly for T2DM, where prolonged morbidity amplifies disability. The T1DM incidence increase, despite mortality and DALY reductions, suggests improved survival rather than a true rise in new cases, possibly due to better diagnostic capabilities identifying previously undetected cases.

The disparity between incidence and mortality trends reflects a critical public health paradox: while survival improves, the sheer volume of new cases—especially T2DM—overwhelms health systems, increasing the chronic disease burden. This necessitates a dual focus on prevention (to curb incidence) and management (to mitigate DALYs and mortality) (9).

### 4.2 Geospatial disparities in diabetes burden

#### 4.2.1 Incidence hotspots: pacific islands vs. low-incidence regions

The age-standardized incidence rate (ASIR) of DM exhibits stark geographic variation. Pacific Island nations—Marshall Islands (884.21), American Samoa (863.36), and Fiji (838.06)—report rates exceeding 800/100,000, dwarfing those in Costa Rica (2.37), China (2.67), and Colombia (2.68). This disparity likely reflects genetic predisposition, rapid nutritional transitions, and limited healthcare infrastructure in Pacific regions (10). High rates of obesity, linked to diets rich in processed carbohydrates and low physical activity, amplify T2DM risk, which dominates these rankings (11, 12).

Conversely, low-incidence nations like China benefit from historically lower obesity rates and dietary patterns emphasizing plant-based foods, though urbanization is eroding these advantages. T1DM’s northern latitude predominance (Finland: 44.40; Canada: 35.24; Italy: 26.70) aligns with autoimmune hypotheses, where environmental triggers (e.g., viral infections) and genetic factors (e.g., HLA haplotypes) cluster in colder climates.

#### 4.2.2 Mortality and DALY extremes

Fiji emerges as a mortality epicenter, with an ASDR of 266.11 for DM—over 5,000% higher than Singapore’s 0.047. T2DM drives this burden (ASDR 265.22), with T1DM mortality mirroring this extreme (265.22). DALYs in Fiji (7,387.94 for DM; 7,322.65 for T2DM) dwarf those in France (354.54) and Belarus (307.04). These figures suggest a catastrophic convergence of high incidence, poor disease control, and limited access to care in Pacific Islands, compounded by socioeconomic constraints. Singapore’s low mortality reflects robust healthcare systems, aggressive screening, and lifestyle interventions, offering a model for mitigation. T1DM-specific DALY extremes in Haiti (180.67) and Finland (120.43) highlight differential burdens: Haiti’s reflects inadequate management in a low-resource setting, while Finland’s, despite high incidence, indicates effective care reducing long-term disability (13).

### 4.3 Temporal trends across socio-demographic index levels

#### 4.3.1 Incidence dynamics

From 1990 to 2021, DM incidence rose globally, with females showing a steeper climb across all subtypes. High-SDI regions saw significant increases in male DM and T2DM cases, and both genders for T1DM, reflecting aging populations and lifestyle shifts. Middle-SDI regions mirrored this for male T2DM, driven by industrialization and dietary westernization. Low-SDI regions, despite lower absolute increases, bear the highest DALY burdens, suggesting a lag in preventive infrastructure. The gender disparity—females outpacing males—may relate to higher obesity rates among women in certain regions, hormonal influences (e.g., postmenopausal insulin resistance), or differential healthcare-seeking behaviors. High-SDI regions’ T1DM rise could reflect improved diagnostics rather than true incidence growth, while T2DM’s escalation tracks closely with obesity and urbanization.

#### 4.3.2 DALY and mortality patterns

T1DM DALYs declined globally, with females showing a steeper drop, likely due to better management reducing complications like nephropathy. T2DM DALYs, however, rose sharply, especially among males, reflecting prolonged survival with comorbidities (e.g., cardiovascular disease). Low-SDI regions consistently exhibit the highest DALYs, driven by limited treatment access and higher complication rates. Mortality trends reinforce this dichotomy: T1DM mortality fell by 31% over 31 years, a testament to therapeutic advances, while T2DM mortality remained stable, with Low-SDI regions sustaining elevated rates. High-SDI regions’ lower mortality reflects superior healthcare, while Middle-SDI areas show intermediate outcomes, balancing rising incidence with improving care.

### 4.4 Age-stratified patterns in 2021

DM and T2DM incidence peak at 59–69 years, forming a spindle-shaped curve typical of age-related metabolic decline and cumulative lifestyle risks. T1DM’s bimodal distribution—lower rates at 50–79 years—reflects its autoimmune etiology, with peaks in childhood (new diagnoses) and older age (survivors or late-onset cases).

DM and T2DM DALYs escalate with age, mirroring progressive disability from complications (e.g., neuropathy, retinopathy). T1DM’s peak at 40–44 years suggests a critical window of disability in early-middle adulthood, possibly from cardiovascular or renal sequelae outpacing mortality reductions.

DM and T2DM mortality rise monotonically with age, driven by comorbidities and frailty. T1DM’s biphasic pattern—rising from 5–44 years, then accelerating at 45–95—reflects early risks (e.g., hypoglycemia) and later cardiovascular mortality, highlighting distinct lifespan vulnerabilities.

### 4.5 Risk factor analysis

Metabolic (e.g., high fasting plasma glucose) and environmental (e.g., temperature) risks dominate T1DM DALYs. Low temperatures historically posed risks in colder regions, declining by 2021 due to better living conditions, while high temperatures rose in Middle- and Low-middle-SDI areas, possibly exacerbating dehydration or infection triggers (14). The positive correlation between T1DM presentation and colder temperatures may be explained by factors such as viral infections (15).

T2DM’s risk triad—metabolic (glucose, BMI), behavioral (diet, smoking), and environmental (pollution)—shows BMI’s alarming rise globally, reflecting obesity’s role as a primary driver (16). Declines in processed meat, smoking, and household pollution indicate partial success in behavioral interventions, though ambient pollution’s increase in lower-SDI regions signals an emerging threat. A meta-analysis demonstrated that meat consumption, especially processed meat and unprocessed red meat, is a risk factor for the occurrence of T2DM in different populations (17). A clinical trial published in The Lancet found that significant weight reduction (>15% to 20%) through dietary substitution and behavioral changes can effectively alleviate T2DM (18).

## 5 Conclusion

The 2021 DM burden reflects a global crisis marked by T2DM’s dominance, geospatial inequities, and rising incidence. While T1DM shows progress, T2DM’s trajectory demands urgent action—targeting obesity, enhancing healthcare access, and addressing environmental risks. These insights underscore the need for tailored, evidence-based strategies to curb this escalating epidemic.

This study is a secondary analysis based on the GBD database; however, GBD estimates often overestimate real-world epidemiological data. Therefore, the interpretation and application of secondary analysis results derived from GBD data require caution. The GBD study relies on epidemiological data reported by individual countries, but diabetes surveillance systems in some low-income countries (particularly Pacific Island nations and African regions) remain underdeveloped, potentially leading to underestimation of incidence and mortality. For instance, diabetes is frequently underreported on death certificates, with cardiovascular diseases listed as the immediate cause of death. Secondly, while this study focuses on metabolic, behavioral, and environmental risks, it does not thoroughly explore the impact of social determinants—such as healthcare accessibility, education levels, and food security—on diabetes management. For example, the high burden in Pacific Island nations may be linked to colonial history-driven nutritional transitions and healthcare system fragility, but these complex contextual factors were not quantified. Additionally, synergistic or antagonistic interactions between risk factors (e.g., obesity combined with vitamin D deficiency) were not systematically analyzed, potentially underestimating comprehensive risks in certain populations.

## Data Availability

The original contributions presented in the study are included in the article/**Supplementary Material**. Further inquiries can be directed to the corresponding author.
