# Supplementary material for: Global burden of diabetes mellitus 1990–2021: epidemiological trends, geospatial disparities, and risk factor dynamics
Source: Front Endocrinol (Lausanne). 2025 Jul 1;16:1596127. doi: 10.3389/fendo.2025.1596127 (PMC12259457; doi:10.3389/fendo.2025.1596127)
Supplement: Supplementary file 1 [file Table1.doc]

**Table 1 Incidence, deaths and DALYs of diabetes in 2021, and percentage change of age-standardized rates by GBD region**

| **All diabetes mellitus** | | | | | | | | | | | | |
| --- | --- | --- | --- | --- | --- | --- | --- | --- | --- | --- | --- | --- |
| **world region** | **2021** | | **Percentage change in rates, between1990 and 2021** | **2021** | | | **Percentage change in rates, between1990 and 2021** | | **2021** | | | **Percentage change in rates, between1990 and 2021** |
|  | **All age incidence cases No. (95% UI)** | **ASIR per 100,000 (95% UI)** |  | **All age death cases No. (95% UI)** | **ASDR per 100,000 (95% UI)** | |  |  | **All age DALY cases No. (95% UI)** | **ASR per 100,000 (95% UI)** | |  |
| Global | 24442180.036(22643512.775,26301698.294) | 287.312(266.935,308.835) | 71.374(67.819,74.498) | 1656634.526(1537698.765,1759551.410) | | 19.614(18.121,20.828) | | 7.946(0.416,14.630) | 78938587.050(66772200.718,94495830.375) | | 916.250(775.930,1096.148) | 38.176(29.744,47.013) |
| Middle SDI | 7906200.349(7259518.267,8592127.834) | 284.850(263.714,308.668) | 56.207(52.332,59.642) | 590771.921(547719.743,628549.927) | | 23.450(21.656,24.963) | | 10.885(1.095,19.286) | 26994840.621(22994782.215,31972123.938) | | 987.546(843.567,1165.268) | 31.151(23.517,39.054) |
| High-middle SDI | 4210638.636(3852193.769,4588612.877) | 257.178(237.760,277.881) | 62.373(58.576,66.093) | 240405.020(217588.252,259725.715) | | 12.320(11.129,13.318) | | -3.654(-11.140,3.683) | 12702526.290(10329166.661,15529834.763) | | 672.507(542.799,826.872) | 31.724(22.402,40.355) |
| Low-middle SDI | 5341305.235(4937184.531,5762424.655) | 300.195(278.119,323.489) | 83.420(79.104,87.838) | 451875.823(413449.627,490413.725) | | 35.125(32.089,38.193) | | 34.531(19.124,49.943) | 18977988.593(16266056.912,22244570.459) | | 1259.757(1090.068,1465.073) | 49.600(36.818,62.531) |
| Low SDI | 1911017.924(1773051.715,2070409.150) | 246.656(228.796,266.651) | 56.919(52.809,60.558) | 162677.855(146058.000,181915.462) | | 36.757(33.186,40.936) | | 3.312(-8.024,15.665) | 7081302.621(6134409.668,8265798.514) | | 1229.925(1072.614,1422.258) | 15.434(4.503,27.029) |
| High SDI | 5048071.606(4717297.714,5376851.076) | 348.269(327.534,371.120) | 103.387(98.147,108.929) | 208889.422(185860.516,221167.694) | | 9.351(8.447,9.825) | | -30.151(-32.760,-28.051) | 13090831.315(10414703.364,16595733.971) | | 717.463(565.114,912.758) | 38.607(25.313,50.945) |
| Western Sub-Saharan Africa | 734945.548(681867.231,792192.778) | 245.336(227.792,265.476) | 62.651(57.667,67.653) | 44433.705(41039.784,47677.194) | | 12.444(11.491,13.349) | | 20.775(1.997,42.778) | 2774525.737(2358466.715,3299973.871) | | 1246.073(1081.842,1469.111) | 32.514(15.388,52.949) |
| Western Europe | 1444279.259(1336350.963,1562030.702) | 254.171(236.469,273.338) | 77.785(73.129,82.684) | 66210.577(56771.263,76507.521) | | 39.768(34.827,45.098) | | -43.385(-46.690,-40.803) | 4095540.567(3256591.738,5235188.060) | | 514.384(396.851,668.256) | 13.481(0.144,25.921) |
| Tropical Latin America | 763215.313(689492.420,843351.392) | 290.760(263.491,321.375) | 30.318(26.158,34.536) | 91183.077(77228.749,98626.434) | | 8.034(6.969,8.600) | | -15.069(-18.429,-12.305) | 2838612.817(2426405.275,3334249.718) | | 1097.064(939.650,1286.600) | -0.200(-4.973,4.520) |
| Southern Sub-Saharan Africa | 228235.060(210625.445,247394.973) | 330.913(306.132,358.014) | 73.773(65.289,81.321) | 68006.688(62483.420,71544.531) | | 27.174(24.863,28.630) | | 77.599(61.627,96.335) | 1272875.237(1165585.927,1408939.894) | | 2161.943(1980.622,2381.717) | 74.836(61.878,88.987) |
| Southern Latin America | 249628.698(226556.745,269980.236) | 318.198(289.599,344.585) | 90.082(77.199,101.577) | 39536.102(36909.680,42447.240) | | 77.182(72.173,82.607) | | -30.212(-34.007,-26.205) | 650167.384(528979.344,801924.476) | | 763.227(619.146,943.649) | 12.753(1.460,24.907) |
| Southeast Asia | 2338211.752(2156559.401,2530200.222) | 316.045(291.814,341.271) | 73.915(69.362,79.533) | 13434.030(12416.660,14229.997) | | 15.003(13.916,15.874) | | 15.303(0.151,32.221) | 8233948.930(7173647.050,9709718.361) | | 1224.341(1070.342,1440.199) | 31.369(16.745,45.171) |
| South Asia | 5056326.560(4613486.408,5533893.808) | 277.340(254.086,301.618) | 68.467(63.273,73.717) | 198785.358(177131.840,220312.436) | | 33.142(29.547,36.683) | | 33.716(14.411,54.533) | 17771815.360(15048496.458,20916829.636) | | 1153.890(988.457,1345.988) | 45.562(29.710,60.594) |
| Oceania | 67837.722(63643.022,72178.921) | 572.898(540.992,605.401) | 69.570(63.674,75.388) | 425539.594(378602.105,468849.084) | | 32.845(29.273,36.175) | | 6.278(-16.070,36.072) | 308329.367(261513.274,367521.262) | | 3586.467(3065.159,4248.631) | 21.023(-1.746,48.247) |
| North Africa and Middle East | 2786899.036(2575639.502,3009254.316) | 460.656(427.937,497.477) | 136.539(129.681,142.966) | 7417.412(6258.550,8974.980) | | 109.690(93.485,131.342) | | 21.367(1.937,35.726) | 6544241.679(5360910.425,8200515.295) | | 1344.969(1115.768,1670.615) | 65.789(45.636,80.700) |
| High-income North America | 2226588.438(2086207.816,2368531.462) | 450.889(422.887,476.933) | 133.866(122.957,144.903) | 115700.951(102326.496,129680.474) | | 29.286(25.940,32.584) | | -19.191(-21.800,-16.569) | 5511662.696(4358245.232,6922328.541) | | 928.716(738.641,1153.735) | 53.524(39.173,66.322) |
| High-income Asia Pacific | 892437.139(813842.196,970441.491) | 347.015(319.993,375.559) | 73.330(66.960,79.883) | 80437.583(72984.689,85237.335) | | 12.187(11.187,12.844) | | -58.450(-61.988,-54.767) | 2305380.781(1726454.116,3040307.869) | | 639.055(471.809,857.870) | 29.236(13.624,43.507) |
| Eastern Sub-Saharan Africa | 448132.944(419539.143,479810.983) | 179.318(167.838,192.360) | 28.758(25.186,32.446) | 21216.765(17720.042,23429.021) | | 3.959(3.399,4.339) | | -11.217(-22.067,1.468) | 2337188.015(2061598.061,2709346.871) | | 1196.886(1061.849,1370.426) | -7.017(-17.634,5.296) |
| Eastern Europe | 545821.131(486150.426,605980.470) | 194.793(175.206,215.386) | 90.603(85.863,95.993) | 61617.190(54467.532,70074.348) | | 41.888(36.706,47.412) | | 187.418(168.214,208.014) | 2000939.348(1682237.609,2423903.434) | | 600.203(502.674,726.773) | 106.007(99.480,114.230) |
| East Asia | 4184952.591(3774465.882,4629424.262) | 245.225(224.821,267.149) | 50.288(43.873,59.127) | 193735.660(163016.122,227838.535) | | 9.432(7.935,11.038) | | -9.582(-25.802,11.016) | 12375411.666(9581336.550,15767322.930) | | 596.681(460.477,766.078) | 24.271(10.876,36.773) |
| Central Sub-Saharan Africa | 242751.141(222312.399,263059.987) | 267.773(246.950,288.496) | 53.559(46.641,60.303) | 24237.170(19262.820,30120.695) | | 52.010(41.529,63.178) | | -0.550(-23.573,23.792) | 1039292.850(856223.450,1266164.471) | | 1638.127(1359.102,1984.706) | 12.981(-7.365,35.414) |
| Central Latin America | 1038025.280(966686.780,1110839.180) | 387.462(362.007,413.898) | 27.880(25.328,30.917) | 118878.820(106957.746,131506.003) | | 48.473(43.623,53.566) | | 0.387(-8.450,9.993) | 4766808.532(4133293.872,5553445.461) | | 1856.443(1610.988,2158.367) | 12.690(5.893,20.306) |
| Central Europe | 453230.570(418125.885,492121.263) | 277.780(257.364,301.195) | 52.738(48.758,56.388) | 33070.601(30321.822,35665.644) | | 14.195(13.070,15.297) | | 4.461(-3.307,12.741) | 1567051.033(1272370.458,1937804.162) | | 751.553(607.129,933.861) | 23.860(16.093,30.832) |
| Central Asia | 254392.444(238164.314,274136.819) | 255.698(239.387,274.994) | 120.808(110.079,128.840) | 14091.501(12404.919,15832.450) | | 17.379(15.416,19.350) | | 73.882(52.001,96.706) | 803995.199(667147.909,993188.679) | | 899.691(751.720,1108.120) | 91.471(76.109,106.006) |
| Caribbean | 228241.148(213171.788,245111.237) | 440.022(411.509,471.723) | 64.265(58.882,69.931) | 20041.551(17243.043,22930.497) | | 37.087(31.923,42.411) | | -19.121(-29.721,-7.689) | 917868.586(764748.457,1116592.860) | | 1723.247(1435.961,2096.782) | 12.248(1.289,23.972) |
| Australasia | 82467.396(75314.654,89198.656) | 206.152(189.047,221.454) | 68.532(60.997,78.147) | 5035.166(4362.189,5473.239) | | 8.522(7.491,9.204) | | -26.050(-31.532,-20.699) | 233552.964(186345.756,295233.716) | | 471.758(372.203,604.600) | 15.578(4.343,26.978) |
| Andean Latin America | 175560.866(164128.451,187497.374) | 274.607(256.132,293.149) | 95.831(89.456,104.090) | 14025.025(11962.391,16803.499) | | 24.319(20.797,29.121) | | 19.376(-1.148,44.041) | 589378.303(490977.656,719840.025) | | 980.367(816.713,1197.092) | 42.917(24.956,61.339) |
| **Diabetes mellitus type 1** | | | | | | | | | | | | |
| **world region** | **2021** | | **Percentage change in rates, between1990 and 2021** | **2021** | | | **Percentage change in rates, between1990 and 2021** | | **2021** | | | **Percentage change in rates, between1990 and 2021** |
|  | **All age incidence cases No. (95% UI)** | **ASIR per 100,000 (95% UI)** |  | **All age death cases No. (95% UI)** | **ASDR per 100,000 (95% UI)** | |  |  | **All age DALY cases No. (95% UI)** | **ASR per 100,000 (95% UI)** | |  |
| Global | 530859.451(455261.055,622954.933) | 6.980(5.986,8.142) | 22.795(20.232,25.517) | 48511.301(43623.652,53957.724) | | 0.594(0.533,0.662) | | -29.241(-39.016,-22.094) | 3597713.337(3037759.104,4249993.600) | | 44.469(37.568,52.381) | -12.004(-20.523,-5.041) |
| Middle SDI | 130333.129(108744.043,157949.536) | 5.633(4.701,6.829) | 23.501(18.735,28.342) | 13212.026(11787.452,14864.010) | | 0.507(0.453,0.569) | | -27.842(-43.461,-16.226) | 910275.061(772059.051,1074045.276) | | 35.027(29.939,41.177) | -14.132(-27.105,-5.335) |
| High-middle SDI | 75410.846(64454.677,89758.446) | 6.646(5.699,7.759) | 41.416(37.446,45.507) | 5405.984(4927.579,6003.194) | | 0.340(0.312,0.375) | | -47.407(-56.921,-39.031) | 471074.052(383323.865,589335.738) | | 30.837(25.403,38.193) | -22.527(-32.557,-13.247) |
| Low-middle SDI | 126876.882(106180.894,152647.755) | 6.364(5.399,7.559) | 12.096(10.390,13.601) | 14424.380(11677.314,16910.971) | | 0.834(0.669,0.985) | | -17.935(-32.613,-2.622) | 935591.998(784315.218,1099887.464) | | 50.134(41.825,59.083) | -12.553(-25.659,-1.829) |
| Low SDI | 79555.757(68349.801,93861.358) | 5.960(5.187,6.863) | 3.920(2.416,5.407) | 8111.912(6459.364,9788.500) | | 0.886(0.674,1.096) | | -25.020(-37.876,-13.259) | 606970.199(496286.543,726693.993) | | 56.986(46.777,68.079) | -21.282(-31.642,-10.248) |
| High SDI | 118198.232(101910.581,137213.626) | 13.248(11.536,15.210) | 44.588(40.305,49.163) | 0.491(0.474,0.507) | | 7295.679(6917.881,7547.640) | | -41.250(-43.342,-39.324) | 669804.093(533123.593,833403.610) | | 49.926(40.075,61.620) | -5.707(-13.124,0.988) |
| Western Sub-Saharan Africa | 29899.066(24781.865,36744.872) | 5.451(4.646,6.423) | 6.133(4.463,7.453) | 2602.807(1973.314,3345.547) | | 0.623(0.464,0.794) | | -26.527(-40.792,-8.436) | 208418.235(157018.940,256888.570) | | 43.855(34.794,54.008) | -18.526(-31.506,-1.985) |
| Western Europe | 58063.141(50333.120,67941.401) | 16.102(14.038,18.505) | 71.541(65.355,78.216) | 1923.134(1780.804,2016.016) | | 0.273(0.261,0.281) | | -57.298(-58.673,-56.017) | 271129.384(197782.329,361750.131) | | 47.080(34.390,62.903) | 6.163(-8.479,19.210) |
| Tropical Latin America | 21103.968(17554.691,25628.521) | 9.819(8.112,12.020) | 27.582(23.751,30.866) | 1653.950(1589.715,1710.534) | | 0.666(0.639,0.690) | | 7.936(2.517,13.321) | 137576.357(115392.168,166801.662) | | 55.639(46.991,67.345) | 17.265(12.362,21.716) |
| Southern Sub-Saharan Africa | 5924.478(4940.280,7151.940) | 7.379(6.235,8.834) | 3.909(1.679,6.064) | 329.124(263.312,376.987) | | 0.408(0.325,0.467) | | -4.634(-19.273,11.870) | 31696.874(25102.063,38314.368) | | 39.551(31.231,48.249) | -3.462(-13.421,6.741) |
| Southern Latin America | 4382.852(3790.813,5011.405) | 7.448(6.363,8.524) | 24.851(15.168,35.492) | 443.317(419.572,469.778) | | 0.552(0.524,0.583) | | -36.807(-40.897,-32.679) | 28735.108(24238.739,34424.654) | | 38.306(32.411,46.338) | -16.443(-22.342,-9.842) |
| Southeast Asia | 50549.656(42263.255,60830.788) | 7.421(6.270,8.923) | 1.109(-1.326,3.415) | 4383.203(3629.370,5790.671) | | 0.608(0.503,0.802) | | -31.263(-51.365,-16.227) | 309247.466(258501.024,378125.568) | | 41.988(35.227,50.996) | -22.073(-40.346,-11.346) |
| South Asia | 116981.986(94599.431,145766.217) | 6.258(5.111,7.749) | 17.480(15.528,19.243) | 15285.510(11588.963,18640.681) | | 0.893(0.670,1.097) | | -20.543(-35.369,-1.106) | 919847.566(753702.281,1104575.273) | | 49.906(40.484,59.872) | -14.993(-27.920,-2.857) |
| Oceania | 953.801(834.343,1092.352) | 6.192(5.530,6.955) | -0.468(-3.842,2.798) | 107.184(78.917,142.137) | | 0.758(0.543,1.020) | | -3.464(-31.235,27.701) | 8291.148(6471.227,10343.270) | | 57.292(44.579,71.831) | -1.748(-23.392,21.495) |
| North Africa and Middle East | 46512.139(39817.646,53631.182) | 7.273(6.268,8.364) | 18.213(14.590,23.129) | 2450.232(2060.892,2964.556) | | 0.407(0.343,0.497) | | -31.328(-47.480,-11.124) | 237792.395(194506.498,289235.556) | | 38.251(31.149,46.526) | -16.532(-30.537,-2.016) |
| High-income North America | 56898.279(48773.818,66485.881) | 17.730(15.229,20.616) | 27.986(22.696,32.842) | 3784.759(3639.879,3898.195) | | 0.838(0.814,0.860) | | -14.322(-16.542,-11.576) | 335060.662(275891.142,409739.716) | | 77.217(64.370,93.340) | 8.645(4.626,13.043) |
| High-income Asia Pacific | 7018.195(5874.103,8450.048) | 6.566(5.320,8.094) | 29.141(21.902,36.086) | 385.134(330.378,467.968) | | 0.137(0.121,0.165) | | -71.218(-75.827,-63.678) | 52520.188(37386.081,69795.462) | | 22.650(16.219,30.470) | -31.716(-44.453,-20.335) |
| Eastern Sub-Saharan Africa | 35742.794(31513.689,40188.302) | 6.628(5.933,7.380) | -0.166(-2.031,1.613) | 3181.688(2479.048,3896.394) | | 0.815(0.613,1.038) | | -31.377(-48.677,-15.867) | 267148.958(217945.216,321688.156) | | 61.785(49.736,74.617) | -26.632(-38.209,-11.744) |
| Eastern Europe | 16163.724(13494.424,19762.258) | 8.851(7.474,10.756) | 54.728(50.926,58.891) | 1359.333(1263.615,1466.720) | | 0.575(0.535,0.617) | | -13.261(-25.061,-3.619) | 119316.306(99016.022,144822.598) | | 50.448(43.012,60.543) | 11.200(0.390,20.032) |
| East Asia | 34367.610(28944.585,42159.461) | 2.747(2.291,3.336) | 38.669(30.634,47.844) | 4665.955(3804.042,5797.814) | | 0.263(0.216,0.323) | | -54.081(-68.497,-36.893) | 280089.488(231754.673,336448.550) | | 16.853(14.032,20.119) | -42.727(-56.188,-27.791) |
| Central Sub-Saharan Africa | 8226.003(7055.827,9500.551) | 5.128(4.530,5.834) | 4.265(0.732,8.127) | 843.848(619.053,1112.628) | | 0.752(0.536,0.997) | | -22.008(-41.045,-0.127) | 62879.093(48721.623,79520.627) | | 49.292(37.730,62.292) | -19.515(-35.552,-0.244) |
| Central Latin America | 11171.453(9197.244,13648.636) | 4.582(3.770,5.617) | -0.455(-7.891,6.646) | 1972.854(1785.763,2160.836) | | 0.757(0.685,0.830) | | -15.545(-24.336,-7.498) | 121324.323(107739.807,138680.017) | | 46.132(40.992,52.780) | -4.111(-11.958,3.055) |
| Central Europe | 8086.793(7009.251,9429.181) | 8.504(7.297,9.969) | 47.360(42.403,52.311) | 1134.884(1024.309,1267.311) | | 0.632(0.573,0.698) | | -56.725(-61.309,-51.598) | 67547.469(56404.928,80613.978) | | 44.219(37.120,52.910) | -31.300(-38.962,-23.710) |
| Central Asia | 8538.246(7546.699,9673.912) | 8.941(7.926,10.157) | 40.574(36.085,46.167) | 920.559(800.660,1051.749) | | 0.976(0.851,1.110) | | -4.540(-18.203,12.846) | 62199.053(53208.051,72820.388) | | 64.228(54.938,75.094) | 10.336(-0.408,22.772) |
| Caribbean | 3737.024(3355.512,4171.320) | 8.370(7.480,9.447) | -0.536(-4.269,3.212) | 798.197(624.041,1039.023) | | 1.593(1.244,2.064) | | -22.484(-37.017,-7.012) | 44585.282(35391.484,54365.375) | | 91.763(72.675,112.727) | -15.092(-27.664,-0.652) |
| Australasia | 4254.720(3824.773,4723.849) | 15.799(14.325,17.533) | 51.692(42.475,60.642) | 108.162(100.005,115.256) | | 0.265(0.247,0.281) | | -51.914(-55.716,-48.327) | 18820.665(14059.286,24811.679) | | 49.431(37.298,64.962) | 7.125(-4.310,18.898) |
| Andean Latin America | 2283.524(1977.565,2647.933) | 3.449(2.988,3.988) | 17.646(12.885,22.470) | 177.466(128.349,227.604) | | 0.271(0.194,0.348) | | -15.194(-35.762,10.621) | 13487.315(10459.232,16599.438) | | 20.247(15.695,24.930) | -8.636(-22.870,7.778) |
| **Diabetes mellitus type 2** | | | | | | | | | | | | |
| **world region** | **2021** | | **Percentage change in rates, between1990 and 2021** | **2021** | | | **Percentage change in rates, between1990 and 2021** | | **2021** | | | **Percentage change in rates, between1990 and 2021** |
|  | **All age incidence cases No. (95% UI)** | **ASIR per 100,000 (95% UI)** |  | **All age death cases No. (95% UI)** | **ASDR per 100,000 (95% UI)** | |  |  | **All age DALY cases No. (95% UI)** | **ASR per 100,000 (95% UI)** | |  |
| Global | 23911320.584(22140751.315,25788088.774) | 280.332(260.061,302.148) | 73.079(69.314,76.490) | 1608123.225(1493437.698,1708293.641) | | 19.020(17.571,20.204) | | 9.747(2.217,16.595) | 75340873.714(63483094.226,90254281.751) | | 871.781(735.055,1044.784) | 42.315(33.696,51.063) |
| Middle SDI | 7775867.220(7132580.361,8475543.837) | 279.216(258.122,302.990) | 57.046(53.038,60.702) | 577559.895(535862.169,614261.379) | | 22.944(21.189,24.401) | | 12.214(2.738,20.709) | 26084565.560(22169178.429,30907857.560) | | 952.519(811.596,1124.097) | 33.744(25.711,41.630) |
| High-middle SDI | 4135227.791(3776896.628,4512465.923) | 250.532(231.085,271.218) | 63.014(59.056,66.891) | 234999.037(212680.338,254106.764) | | 11.981(10.817,12.966) | | -1.325(-8.906,6.184) | 12231452.238(9946570.839,14957461.206) | | 641.671(516.907,788.334) | 36.311(27.056,44.792) |
| Low-middle SDI | 5214428.353(4811617.525,5636512.272) | 293.831(272.023,317.368) | 85.983(81.421,90.454) | 437451.443(399916.749,474443.532) | | 34.290(31.308,37.193) | | 36.657(21.157,52.370) | 18042396.595(15414619.653,21210334.865) | | 1172.939(1026.095,1362.031) | 54.141(40.702,67.159) |
| Low SDI | 1831462.167(1693032.601,1987875.503) | 240.696(222.662,260.538) | 58.926(54.713,62.747) | 154565.943(139065.444,172691.761) | | 35.870(32.300,39.940) | | 4.286(-7.218,16.743) | 6474332.422(5608420.447,7601171.995) | | 1209.623(1045.252,1411.328) | 18.111(6.726,29.824) |
| High SDI | 4929873.374(4603212.811,5266838.351) | 335.022(314.705,358.306) | 106.712(101.131,112.772) | 201593.743(178953.374,213767.743) | | 8.859(7.966,9.328) | | -29.411(-32.099,-27.287) | 12421027.222(9861777.847,15785615.377) | | 667.537(523.843,853.828) | 43.656(29.671,56.851) |
| Western Sub-Saharan Africa | 705046.481(652920.048,761474.074) | 239.885(222.177,259.783) | 64.643(59.454,69.828) | 63607.770(54748.138,73218.195) | | 39.144(34.363,44.323) | | 22.027(2.709,44.076) | 2566107.503(2198106.722,3065834.931) | | 1202.217(1043.855,1417.681) | 35.614(17.860,56.556) |
| Western Europe | 1386216.118(1278807.217,1504845.918) | 238.069(220.382,257.373) | 78.223(73.322,83.667) | 89259.943(75435.013,96635.613) | | 7.762(6.709,8.320) | | -42.730(-46.171,-40.083) | 3824411.183(3049988.126,4885188.460) | | 467.304(362.809,608.131) | 14.275(0.996,27.001) |
| Tropical Latin America | 742111.345(669137.672,823488.899) | 280.942(253.900,311.216) | 30.416(26.147,34.766) | 66352.738(60865.672,69850.851) | | 26.508(24.213,27.961) | | -15.521(-18.871,-12.712) | 2701036.460(2302968.593,3184550.388) | | 1041.425(889.700,1225.380) | -0.988(-5.829,3.836) |
| Southern Sub-Saharan Africa | 222310.583(204643.080,241937.657) | 323.534(298.957,350.135) | 76.480(67.468,84.488) | 39206.978(36588.336,42074.850) | | 76.774(71.789,82.157) | | 78.417(62.390,97.252) | 1241178.363(1137268.953,1372925.644) | | 2122.392(1946.587,2338.019) | 77.519(64.365,92.082) |
| Southern Latin America | 245245.846(221936.276,266022.273) | 310.750(281.937,336.990) | 92.492(79.165,104.125) | 12990.713(11989.191,13774.769) | | 14.451(13.382,15.303) | | -29.932(-33.868,-25.770) | 621432.275(503705.365,771130.035) | | 724.920(585.949,900.905) | 14.874(3.000,27.544) |
| Southeast Asia | 2287662.096(2107044.819,2478966.309) | 308.624(284.784,333.749) | 76.979(72.117,83.041) | 194402.155(173155.247,215512.067) | | 32.534(28.997,36.002) | | 16.783(1.749,34.003) | 7924701.464(6898462.719,9360596.422) | | 1182.353(1033.183,1393.525) | 34.648(20.492,49.249) |
| South Asia | 4939344.574(4503489.419,5418417.630) | 271.082(247.808,295.778) | 70.172(64.853,75.683) | 410254.084(364228.649,452153.733) | | 31.953(28.465,35.189) | | 36.316(16.451,57.431) | 16851967.793(14173188.127,19903034.206) | | 1103.984(940.980,1291.723) | 50.406(34.278,65.045) |
| Oceania | 66883.921(62730.887,71292.876) | 566.706(534.570,599.168) | 70.884(64.832,76.777) | 7310.228(6170.987,8863.842) | | 108.932(92.843,130.511) | | 6.352(-16.012,36.232) | 300038.219(254617.478,356814.566) | | 3529.175(3015.852,4180.853) | 21.480(-1.391,48.779) |
| North Africa and Middle East | 2740386.897(2529649.153,2963580.632) | 453.383(420.733,489.865) | 140.399(133.003,147.380) | 113250.719(100173.924,126911.275) | | 28.878(25.567,32.137) | | 22.694(2.901,37.204) | 6306449.285(5148287.225,7933908.808) | | 1306.718(1081.868,1628.682) | 70.718(49.888,86.586) |
| High-income North America | 2169690.159(2030617.657,2313515.009) | 433.159(406.146,459.946) | 142.063(129.897,155.013) | 76652.824(69349.353,81349.647) | | 11.349(10.373,12.002) | | -19.529(-22.283,-16.759) | 5176602.034(4078249.690,6518944.348) | | 851.498(670.238,1068.288) | 59.499(43.306,73.529) |
| High-income Asia Pacific | 885418.945(806657.618,963482.627) | 340.449(313.611,368.776) | 74.481(67.881,81.232) | 20831.631(17389.258,23032.915) | | 3.822(3.278,4.204) | | -57.781(-61.271,-54.054) | 2252860.593(1682408.797,2972522.551) | | 616.405(454.540,828.046) | 33.619(17.788,48.013) |
| Eastern Sub-Saharan Africa | 412390.150(383582.973,442747.510) | 172.689(161.185,185.679) | 30.206(26.436,34.086) | 58435.502(51367.283,66716.235) | | 41.073(36.003,46.517) | | -10.697(-21.800,2.202) | 2070039.057(1830453.481,2386737.990) | | 1135.101(1005.599,1293.465) | -5.644(-16.811,6.992) |
| Eastern Europe | 529657.407(471244.594,590679.793) | 185.942(165.642,206.726) | 92.730(87.882,98.520) | 43074.372(39768.829,46225.208) | | 11.869(10.952,12.737) | | 223.681(201.384,247.106) | 1881623.042(1578549.084,2284952.677) | | 549.755(457.582,669.279) | 123.492(114.787,135.333) |
| East Asia | 4150584.982(3740755.683,4598029.569) | 242.478(222.153,264.162) | 50.431(43.890,59.410) | 189069.705(159223.747,222659.583) | | 9.169(7.715,10.748) | | -6.998(-23.567,14.229) | 12095322.178(9347450.478,15426635.174) | | 579.828(444.761,744.644) | 28.645(16.151,40.876) |
| Central Sub-Saharan Africa | 234525.138(214621.638,254707.284) | 262.644(241.966,283.419) | 54.990(47.861,61.879) | 23393.322(18505.934,29119.532) | | 51.258(40.927,62.335) | | -0.146(-23.213,24.468) | 976413.757(803272.843,1198184.072) | | 1588.835(1318.317,1924.419) | 14.414(-6.072,37.817) |
| Central Latin America | 1026853.827(955757.125,1099920.370) | 382.880(357.102,409.477) | 28.317(25.758,31.486) | 116905.966(105170.440,129323.950) | | 47.716(42.939,52.726) | | 0.688(-8.181,10.314) | 4645484.208(4025907.964,5420647.808) | | 1810.311(1569.507,2107.780) | 13.195(6.330,20.875) |
| Central Europe | 445143.778(410134.647,483749.303) | 269.275(249.269,292.397) | 52.914(48.841,56.651) | 31935.716(29245.589,34482.429) | | 13.563(12.461,14.630) | | 11.823(3.481,20.713) | 1499503.564(1213156.929,1860340.950) | | 707.334(569.372,884.839) | 30.406(23.284,36.805) |
| Central Asia | 245854.197(229544.946,265389.149) | 246.757(230.694,265.869) | 125.471(113.726,133.947) | 13170.942(11590.968,14803.996) | | 16.403(14.548,18.234) | | 82.818(59.469,107.848) | 741796.146(613112.975,920193.178) | | 835.463(694.556,1033.015) | 102.943(87.183,118.748) |
| Caribbean | 224504.124(209571.121,241386.111) | 431.652(403.600,463.625) | 66.367(60.826,72.141) | 19243.354(16584.338,22068.726) | | 35.494(30.602,40.688) | | -18.963(-29.717,-7.544) | 873283.304(726985.367,1063097.416) | | 1631.484(1357.700,1987.215) | 14.318(3.258,26.075) |
| Australasia | 78212.676(71050.757,84992.315) | 190.352(173.081,205.362) | 70.099(61.858,80.531) | 4927.005(4257.464,5367.155) | | 8.257(7.237,8.930) | | -24.750(-30.524,-19.157) | 214732.298(171656.629,268249.371) | | 422.327(336.034,531.227) | 16.655(4.641,28.104) |
| Andean Latin America | 173277.341(161976.518,185204.026) | 271.158(252.574,289.886) | 97.501(90.988,106.055) | 13847.559(11827.522,16602.054) | | 24.047(20.583,28.783) | | 19.927(-0.793,44.511) | 575890.988(478727.284,703763.892) | | 960.120(799.892,1171.011) | 44.638(26.293,63.672) |
